# Consensus Recommendations to Optimize the Detection and Reporting of *NTRK* Gene Fusions by RNA-Based Next-Generation Sequencing

**DOI:** 10.3390/curroncol30040302

**Published:** 2023-03-31

**Authors:** Tracy L. Stockley, Bryan Lo, Adrian Box, Andrea Gomez Corredor, John DeCoteau, Patrice Desmeules, Harriet Feilotter, Daria Grafodatskaya, Cynthia Hawkins, Weei Yuarn Huang, Iyare Izevbaye, Guylaine Lepine, Andreas I. Papadakis, Paul C. Park, Brandon S. Sheffield, Danh Tran-Thanh, Stephen Yip, Ming Sound Tsao

**Affiliations:** 1 Laboratory Medicine Program, University Health Network, Toronto, ON M5G 2C4, Canada; 2 Department of Laboratory Medicine and Pathobiology, University of Toronto, Toronto, ON M5R 0A3, Canada; 3 Advanced Molecular Diagnostics Laboratory, Princess Margaret Cancer Centre, Toronto, ON M5G 2C1, Canada; 4 Department of Pathology and Laboratory Medicine, The Ottawa Hospital, Ottawa, ON K1H 8L6, Canada; 5 Alberta Precision Laboratories, Calgary, AB T2N 7A4, Canada; 6 McGill University Health Center, Montreal, QC H4A 3J1, Canada; 7 Department of Pathology and Laboratory Medicine, College of Medicine, University of Saskatchewan, Saskatoon, SK S7N 5A9, Canada; 8 IUCPQ-UL, Quebec Heart and Lung Institute, Quebec City, QC G1V 4G5, Canada; 9 Kingston Health Sciences Centre, Kingston, ON K7L 2V7, Canada; 10 Department of Pathology and Molecular Medicine, Queens University, Kingston, ON K7L 3N6, Canada; 11 Hamilton Health Sciences Centre, Hamilton, ON L9C 1C4, Canada; 12 Department of Pathology and Molecular Medicine, McMaster University, Hamilton, ON L8S 4K1, Canada; 13 Department of Paediatric Laboratory Medicine, The Hospital for Sick Children, Toronto, ON M5G 1X8, Canada; 14 Nova Scotia Health Authority, Halifax, NS B3H 2E2, Canada; 15 Sunnybrook Health Sciences Centre, Toronto, ON M4N 3M5, Canada; 16 Department of Laboratory Medicine and Pathology, University of Alberta, Edmonton, AB T6G 2R7, Canada; 17 Hopital Maisonneuve-Rosemont, Montreal, QC H1T 2M4, Canada; 18 Lady Davis Institute, Jewish General Hospital, Montreal, QC H3T 1E2, Canada; 19 Shared Health Manitoba, Winnipeg, MB R3A 1R9, Canada; 20 William Osler Health System, Brampton, ON L6R 3J7, Canada; 21 CHUM—Centre Hospitalier de l’Universite de Montreal, Montreal, QC H3T 1J4, Canada; 22 BC Cancer, Vancouver, BC V5Z 4S6, Canada; 23 Department of Pathology and Laboratory Medicine, University of British Columbia, Vancouver, BC V6H 3Z6, Canada

**Keywords:** next generation RNA sequencing, molecular pathology, oncology, NTRK gene fusions

## Abstract

The detection of gene fusions by RNA-based next-generation sequencing (NGS) is an emerging method in clinical genetic laboratories for oncology biomarker testing to direct targeted therapy selections. A recent Canadian study (CANTRK study) comparing the detection of *NTRK* gene fusions on different NGS assays to determine subjects’ eligibility for tyrosine kinase TRK inhibitor therapy identified the need for recommendations for best practices for laboratory testing to optimize RNA-based NGS gene fusion detection. To develop consensus recommendations, representatives from 17 Canadian genetic laboratories participated in working group discussions and the completion of survey questions about RNA-based NGS. Consensus recommendations are presented for pre-analytic, analytic and reporting aspects of gene fusion detection by RNA-based NGS.

## 1. Introduction

Next-generation sequencing (NGS) targeted panel testing is routinely used in Canadian clinical molecular diagnostic laboratories for detection of somatic variants that are useful for cancer diagnosis, prognosis and therapy in solid tumors [1]. Somatic tumor NGS tests require the detection of multiple variant types, including single nucleotide variants (SNV), small insertions and deletions, copy number variants and gene fusions in well-defined, clinically relevant genes. However, due to regional differences in expertise, funding and NGS platform availability, there is variability in the specific NGS platforms or assays that are adopted by different clinical laboratories within Canada.

Recently, a Canada-wide project (CANTRK) compared the performance of seven RNA-based NGS targeted panels for the detection of gene fusions involving neurotrophic tyrosine receptor kinase genes (*NTRK1*, *NTRK2* and *NTRK3*) in 16 diagnostic clinical laboratories across Canada [2]. NGS assays included four different amplicon-based panels, one hybrid-capture panel, one anchored multiplex PCR panel and one single primer extension and amplification panel. Twelve formalin-fixed paraffin-embedded tumor samples containing a gene fusion involving one of *NTRK1*, *NTRK2* or *NTRK3*, with six different fusion gene partners, were tested by each laboratory. The findings of the comparative study indicated that eight of the twelve samples were detected by all NGS Panels. Three of four amplicon NGS panels did not detect two fusions involving *NTRK2* (fusions *WNK2: NTKR2* and *STRN3: NTRK2*) due to absence of primers in the panel design. For one amplicon panel, two *NTRK1* fusions (*TPM3: NTRK1* and *LMNA: NTRK1*) were not detected due to bioinformatic challenges. The one hybrid capture panel did not detect an *NTRK3* fusion, *ETV6: NTRK3*, found at low levels in one sample. Overall, the CANTRK project indicated good but variable ability of the different RNA-based NGS panels implemented in Canadian laboratories to detect *NTRK* gene fusions [2].

It was also evident during the CANTRK study that there was limited consensus guidance on how to best to perform gene fusion detection for *NTRK*, or other fusion genes, using the emerging method of RNA-based NGS. Several publications have addressed the topic of diagnostic test algorithms for detecting *NTRK* gene fusions or TRK fusion proteins, using methods such as immunohistochemistry, fluorescence in situ hybridization, reverse transcriptase-polymerase chain reaction (RT-PCR), and DNA or RNA-based NGS [3,4,5,6,7,8,9,10]. Other publications have described the validation or the clinical implementation of NGS panels capable of detecting gene fusions in solid tumors or hematologic malignancies, providing useful information on defining quality metrics, performance characteristic assessment, verification of novel fusions and troubleshooting [11,12,13,14,15,16]. However, testing for gene fusions using RNA-based NGS is based on different principles than for DNA-based NGS. The output of RNA-based NGS for gene fusions is only positive when gene fusions are present, and so a negative result (i.e., no gene fusions) is a common occurrence, and quality metrics used in DNA-based NGS testing, such as variant allele fraction or read depth, do not have direct equivalents in RNA-based NGS. For this reason, best practices are needed for RNA-based NGS for detection of gene fusions. As a partner activity to the CANTRK study, and to address this need in the Canadian context, a national Canadian clinical laboratory expert group convened to share approaches to gene fusion detection using RNA-based NGS and to develop consensus recommendations relevant to national laboratories to ensure high-quality detection of *NTRK* and other gene fusions from RNA-based NGS.

## 2. Materials and Methods

An expert working group of eighteen Canadian clinical molecular laboratory directors and pathologists was formed, with members representing sixteen participating CANTRK study laboratories plus one additional Canadian laboratory and seven provinces (Nova Scotia, Quebec, Ontario, Manitoba, Saskatchewan, Alberta and British Columbia). The aim of the group was to develop guidance for gene fusion detection in solid tumors using RNA-based NGS on formalin-fixed, paraffin-embedded (FFPE) tumor tissue, with a focus on NGS assays used in Canada [2]. All participants had experience with RNA-based NGS in their associated laboratories, whether from the CANTRK study testing or from routine clinical testing in their laboratories. The expert working group members completed pre-meeting surveys (Supplementary Table S1) and met virtually to discuss and reach a consensus on the findings of the survey, spanning the testing pathway from pre-analytic and analytic phases to clinical reporting.

## 3. Pre-Analytic Considerations

The working group consensus recommendations addressing specific pre-analytic activities relevant to NGS RNA-based fusion detection are shown in Table 1, with discussion points following.

### 3.1. Tissue Treatment and Tumor Enrichment

As reported in the CANTRK study, local pre-analytical practices for gene fusion detection by NGS varied based on the specific RNA-based NGS assay or platform used [2]. Most laboratories co-extracted DNA and RNA from the same tumor sample. The enrichment of tumors prior to NGS was performed among the CANTRK participant laboratories by macrodissection of FFPE tissue to enrich for tumor material prior to extraction, as indicated by 71% (12/17) of respondents (Supplementary Table S1), versus the non-enrichment techniques used in the remaining five labs (such as RNA extraction from FFPE scrolls without enrichment). Diverse enrichment techniques introduce variability to pre-analytical workflows, and the working group consensus was that approaches to minimize variability among samples tested in the same laboratory are essential. In some cases, technology may help reduce variability, for example, by using automated macrodissection systems, which mitigate user-variation by documenting the workflow and capturing images before and after enrichment. Decisions regarding tumor enrichment should be based on the particular NGS assay used by each laboratory and the required lower limit of sensitivity of the assay. Any variability also carries over to the assessment of tumor cellularity due to differences in estimation (i.e., over- or underestimation), which can affect the evaluation of borderline samples. In addition, tumor cellularity estimates may be impacted by how far from the hematoxylin and eosin-stained slide the sections are taken for DNA/RNA extraction, and the tumor cell count may be a poor surrogate for potential DNA or RNA quantity due to tumor cell aneuploidy.

The discussion of optimal approaches for macrodissection enrichment included the proposal of performing a hematoxylin and eosin staining on a final section after macrodissection to ensure that the target tissue was obtained. If possible, macrodissection was preferred to the coring of tumor tissue in an FFPE block (using a small 1–2 mm punch) to maintain the integrity of the FFPE block, especially for tumors that were small in volume or size. Reviews were performed by board-certified pathologists in most of the laboratories (94%; 16/17), with a minimum tumor cellularity of 10% or more required in 65% (11/17) of laboratories (Supplementary Table S1).

There was discussion that due to the lability of RNA, laboratories should ensure optimal treatment of tumor tissue prior to FFPE treatment, including monitoring the pre-ischemic time for formalin fixation and maintaining ideal storage and transportation conditions for FFPE blocks. It was noted that international standards exist as a reference for pre-analytic considerations for RNA extraction from FFPE tumor tissue [17].

There was a consensus that, in order to understand the impact of tumor cellularity and necrosis on assay sensitivity and downstream testing, a critical aspect of pre-analytic variability was appropriate education and training of pathologists who select tumor tissue for enrichment. Continuing education for laboratory technologists to raise their awareness of the importance of proper tumor tissue fixation and embedding practices is also essential to minimize pre-analytic variability or failures. Laboratories may also wish to restrict the selection of tumor regions for macrodissection to specific pathologists to mitigate variability, although this is not feasible in every laboratory or pathology department, depending on pathologists’ expertise, work distribution and roles.

### 3.2. Input RNA for RNA-Based NGS Testing

For RNA input requirements, 65% (11/17) of respondents used 10–30 ng of RNA (cDNA), likely reflecting the widespread use of amplicon library methods in Canadian laboratories at the time of the survey and as previously reported [2]. It was noted that different RNA quantification methods are also a potential source of pre-analytic variability. Most participants (71%; 12/17) stated that for challenging tumor specimens where no alternative tissue specimen existed, they would proceed by testing input RNA amounts at the lower validated limit of their assay, and they may report any finding from such testing with report caveats for negative samples or perform complementary testing (such as TRK IHC) if required to confirm a positive result. The consideration to use input RNA below a lab-defined threshold reflected the biological difference in the detection of gene fusions using RNA rather than DNA, as gene fusion drivers may be expressed at high levels, thus, allowing for their detection even with reduced RNA input. In addition, there was discussion among the participants that tumor cellularity estimates are not always accurate for RNA-based assays, as the amount of the RNA fusion does not necessarily relate to the amount of tumor present in the tissue due to the impact of the RNA expression on each specific target fusion.

## 4. Analytic and Interpretive Considerations

The working group consensus recommendations addressing analytic and interpretive aspects of RNA-based NGS for gene fusion detection are shown in Table 2.

### 4.1. Quality Control during Library Preparation

During the NGS library and sequencing workflow, there are multiple points for the capture of quality metrics to assess the overall process and any detected gene fusions. While the majority of quality control metrics are platform-dependent, recommended activities include the assessment of cDNA size, quantity and/or quality prior to use in library preparation (8 of 17 laboratories indicated doing so by either measuring the cDNA size and quantity or by doing the previous and additionally ensuring the cDNA quality by amplifying a housekeeping gene; Supplementary Table S1). It is also recommended to use internal and/or external controls to ensure the detection of target genes (eight laboratories used both internal and external controls, with the remainder of the laboratories using one type of control; Supplementary Table S1).

### 4.2. Quality Control for Gene Fusion Calling

A critical aspect of quality control for gene fusion detection by RNA-based NGS is ensuring that any gene fusions detected are true fusions and not arising from artifacts. This is particularly true for NGS methods using amplicon libraries, where the amplification method produces a greater number of transcripts containing the fusion and may produce artifacts such as misaligned transcripts. The participants used a variety of quality metrics to assess the detected gene fusions and to determine if they were true fusions; the most frequently recommended metrics included:

- A minimum number of supporting reads spanning the gene fusion junction;
- A minimum percentage of supporting reads spanning the fusion gene (percentage calculated as fusion reads compared to total mapped reads);
- A minimum number of unique start sites, where a subset of the unique reads has unique fragment lengths;
- A minimum number of base pairs on either side of the fusion breakpoint.

The participants also discussed the importance of ensuring that negative results (no fusion detected) were valid, which was mainly assessed by ensuring a minimum number of total reads after sequencing.

Once a detected gene fusion was confirmed to meet quality metrics and, so, was deemed a reliable call, laboratories used a variety of approaches to further inspect and confirm the fusion. The initial assessment was to determine if the gene fusion was previously known or was novel, which was achieved by searching the detected fusion in published literature or in relevant databases (such as Quiver Fusion Database [18], CIViC [19], TumorFusions portal [20] and FusionGDB [21]). Other assessments included the requirements that fusions should generate productive in-frame transcripts, have non-disrupted important functional domains (such as the kinase domain in *NTRK* genes [22]) and, where relevant, involve the concordance of variants seen on DNA with RNA fusions in the same sample (for example, *MET* gene fusions of exons 13 and 15, which can also often be detected by *MET* exon 14 splice variants on DNA-based NGS). In addition, it was noted that when two gene fusions are detected in the same sample that are similar but differ by only one exon, only the most abundant transcript was reported in 59% (10/17) labs. For novel gene fusions, it was recommended to use a follow-up orthogonal assay, such as amplification with gene-specific primers flanking the fusion junction and Sanger sequencing, immunohistochemistry or FISH. It is imperative that the appropriate orthogonal assay is applied to the relevant tissue type; for example, immunohistochemistry for an *NTRK* gene fusion is not appropriate in central nervous system tumors due to the known intrinsic expression of TRK proteins, which prevent the discrimination of *NTRK*-rearranged and non-rearranged tissue, in this tissue.

There was agreement regarding the benefit of using controls to confirm appropriate test performance, although no consensus was reached on the type of control or how often the controls should be assayed. The discussion included the utility of internal controls, such as the detection of housekeeping genes included in the assay, and external controls, such as reference standard samples with known gene fusion variants. It was recommended that at a minimum, external control samples should be used routinely to confirm the reliable detection of target gene fusions by the assay. Some participants noted that they use controls on a rotational basis, such as when there is a reagent lot change in the laboratory workflow.

## 5. Post-Analytic and Reporting Recommendations

The working group consensus recommendations addressing post-analytic and reporting of RNA-based NGS for gene fusion detection are shown in Table 3.

With respect to the clinical reports generated from RNA-based NGS, all participant laboratories include an interpretive statement regarding the clinical relevance of gene fusions deemed to meet quality metrics for inclusion in the report. For ease of report understandability, participants suggested that interpretive statements regarding NGS-detected fusions should document clinically relevant information in a simplified section at the top of the report, with additional supporting data presented later in the report if needed.

For gene fusion nomenclature, recent HUGO guidelines recommend the use of a double-colon separator for describing gene fusions (e.g., *BCR*: *ABL*) [23]. There was a consensus that fusion nomenclature should also include the exons of the fusion partners, with reference sequences (mRNA transcript numbers and human reference genome sequence). While reporting exons for gene fusion may be clinically relevant for some fusions (for example, *ALK* fusions) and not for others, it was considered that it is more appropriate to implement exon reporting in general practice, particularly for clarity in the use of the information. Additional information for clinical reports included quality metrics such as the total number of reads, the number or percentage of reads spanning the fusion junction and the minimum number of unique start sites for reads containing a fusion.

For gene fusions tested on panels with both DNA and RNA library analysis, detection of variants on DNA and RNA is possible. For example, mutations in the intron-exon boundaries of *MET* exon 14, which may be detectable at the DNA level, can give rise to gene transcripts where *MET* exon 13 and exon 15 are fused, with the resultant gene fusion also detectable at the RNA level. In this scenario, the majority of the participant laboratories (71%; 12/17) stated that they reported both the DNA variant and the resulting RNA fusion.

Participants were agreed that variant classification schemes for RNA-based NGS assays should be same as the schemes currently in use for DNA variants, such as the scheme reported in [24]. In the system of [24], known or previously reported gene fusions with clinical actionability should be classified as strongly clinically actionable (i.e., Tier I). Novel fusions that have not been reported previously, or for which there is no known prognostic or therapy-related information, should be reported as either Tier II (potentially clinically actionable) or Tier III (unknown clinical significance) depending on the particular genes involved in the fusion.

Molecular laboratory reporting of gene fusion detection by NGS was integrated into an overall pathology report for all tumor histologies tested in 41% (7/17) of participant laboratories and only integrated for certain tumor histologies in 24% (4/17) of laboratories. There was no consensus among the participants on how an integrated report should be formulated, and there was a concern that a lack of training in integrating molecular results into pathology reports could lead to misinterpretations or misinformation on reports, highlighting the importance of educating pathologists on NGS and interpretation of NGS results. Finally, the group discussed the importance of participating in external quality assurance (EQA) programs specific to gene fusion detection by NGS assays as a benchmark to ensure optimal test performance.

## 6. Discussion

Canadian molecular diagnostic laboratories are increasingly using targeted paired library DNA- and RNA-based NGS assays to detect somatic tissue variants in solid tumors, including for the detection of gene fusions of high clinical actionability, such as *NTRK*, *ALK*, *RET* and other fusions. The significant clinical impact of the detection of such fusions requires that clinical laboratories use optimal approaches to detect all fusions, a need which is complicated by the current lack of standardization on how best to perform RNA-based NGS for fusion detection. As a companion activity to the CANTRK Canadian study to optimize detection of *NTRK* gene fusions [2], we report on the discussion and survey results of the 17 CANTRK participant laboratories regarding the current practices and consensus for RNA-based NGS. The understanding of laboratory practices for quality metrics, detection of fusions and assessment of clinical impact provide information that can be used to standardize practices across Canada.

## Figures and Tables

**Table 1:** Summary of pre-analytical recommendations to optimize RNA-based NGS for gene fusion detection.

I	If tumor enrichment is used for samples to be analyzed for gene fusions by RNA-based next generations sequencing (NGS), laboratories should ensure standardization of pre-analytic technical procedures and minimized variability among samples.
II	Decisions regarding use of tumor enrichment should be based on the particular RNA-based NGS assay used and the required lower limit of sensitivity of the assay to detect gene fusions.
III	If macrodissection is used for tumor enrichment, a hematoxylin and eosin-stained section taken after macrodissection should be assessed to ensure that target tissue was obtained for NGS testing.
IV	Due to the lability of RNA in tissue, pre-analytic treatment of tissue and formalin-fixed, paraffin-embedded (FFPE) blocks should be optimized to ensure that extraction of RNA is appropriate for testing for gene fusions (e.g., monitoring pre-ischemic time for tissue formalin fixation, ensuring optimal storage and transportation of FFPE tissue materials).
V	Education should be undertaken to ensure that pathology laboratory professionals (pathologists, laboratory technicians and technologists) are informed of the impact of tumor tissue treatments and tumor enrichment on downstream testing using RNA-based NGS.

**Table 2:** Summary of the analytical and interpretive recommendations for RNA-based NGS for gene fusion detection.

I	Laboratories should assess complementary DNA (cDNA) size, quantity and/or quality prior to use in library preparation for RNA-based NGS.
II	Laboratories should use internal and/or external controls to ensure detection of target genes/gene fusions. At minimum, external control samples should be used routinely to confirm reliable detection of gene fusions targeted by the assay.
III	Quality metrics to assess gene fusions detected after NGS testing should be defined by the laboratory. Recommended metrics include: A required minimum number of supporting reads spanning the gene fusion junction; A required minimum percentage of supporting reads spanning the fusion gene (percentage calculated as fusion reads compared to total mapped reads); A required minimum number of unique start sites, where a subset of the unique reads has unique fragment lengths; A required minimum number of base pairs on either side of the fusion breakpoint.
IV	Laboratories should ensure that negative results (no fusion detected) are valid by confirming that a minimum number of total reads were obtained after sequencing.
V	Laboratories should use published literature and gene fusion databases to assess whether an identified gene fusion is novel or previously known.
VI	Laboratories should assess the functional aspects of identified gene fusions (particularly for novel fusions), such as the potential to generate productive in-frame transcripts or the non-disruption of protein functional domains.
VII	For novel gene fusions, additional orthogonal testing may be required to confirm the presence of the gene fusion.

**Table 3:** Summary of post-analytic and reporting recommendations for RNA-based NGS for gene fusion detection.

I	Laboratory reports should include an interpretive statement of the clinical relevance of gene fusions reported.
II	Laboratories should report the exon boundaries of detected gene fusions with reference sequences.
III	Variant nomenclature guidelines for gene fusions should be used, e.g., HUGO guidelines [23].
IV	Laboratories should use variant classification tier schemes to report the clinical significance of gene fusions, with appropriate references for any schemes used included in the report text.
V	Laboratories should participate in external quality assurance programs specific to gene fusion detection by NGS.

## Supplementary Materials

The following supporting information can be downloaded at: https://www.mdpi.com/article/10.3390/curroncol30040302/s1, Table S1: Survey questions and responses from 17 participant laboratories.
