# Supplementary material for: Consensus Recommendations to Optimize the Detection and Reporting of NTRK Gene Fusions by RNA-Based Next-Generation Sequencing
Source: Curr Oncol. 2023 Mar 31;30(4):3989–97. doi: 10.3390/curroncol30040302 (PMC10136625; doi:10.3390/curroncol30040302)
Supplement: Supplementary file 1 [file curroncol-30-00302-s001.zip › curroncol-2274951-supplementary.pdf]

Table S1. Survey questions and responses from 17 participant laboratories. Note that certain questions allowed participants to select more than one response, as indicated by 'choose all that apply' beside these questions.

Prior to Nucleic Acid Extraction from a Tissue Sample, Is Tumor Cellularity Estimated within the Tumor-Rich Area?	N (Total 17)	Responses (%)
Yes, estimated by a staff pathologist	16	94%
No	1	6%
**When receiving a section from formalin-fixed, paraffin-embedded (FFPE) blocks for RNA extraction, does your standard operating procedure require sections to be within a certain distance from the hematoxylin and eosin (H&E) slide sued for the tumor cellularity estimate?**	**N (Total 17)**	**Responses (%)**
No requirements	6	35%
A fresh section is stained with hematoxylin and eosin and reviewed; then sections immediately following are taken for RNA extraction	5	29%
The hematoxylin and eosin-stained slide must be within a specified number of sections of those used for extraction	3	18%
Other	3	18%
**How are FFPE samples most often treated prior to nucleic acid extraction at your site?**	**N (Total 17)**	**Responses (%)**
Macrodissection of tumor-rich region	12	71%
No enrichment of tumor-rich region prior to extraction	4	24%
Other	1	6%
**What sample types has your site validated for the sequencing of RNA libraries for solid tumors by NGS? (Choose all that apply)**	**N (Total 29)**	**Responses (%)**
FFPE tissue	18	62%
Cytology samples (e.g., fresh samples)	5	17%
Fresh frozen tissue	4	14%
Bone biopsy	2	7%
**If your laboratory has a stated lower cut-off limit of tumor cellularity prior to nucleic acid extraction for RNA, please indicate what this lower cut-off is**	**N (Total 17)**	**Responses (%)**
5% tumor cellularity	1	6%
10% tumor cellularity	8	47%
20% tumor cellularity	3	18%
We do not use a lower cut off limit	3	18%
Other (not yet established, or attempt extraction regardless of cellularity)	2	12%
**Do you test samples with tumor cellularity estimates below your stated cut-off (e.g., in situations where no alternative tissues exist)?**	**N (Total 17)**	**Responses (%)**
Yes	12	71%
No	2	12%
Other	3	18%
**What amount of input RNA is required in your cDNA library preparation?**	**N (Total 17)**	**Responses (%)**
10–20 ng	7	41%
20–30 ng	4	24%
40–50 ng	2	12%
Other (RNA not qualified, use a range of input amounts, or use more than 50 ng)	4	24%

Does your lab perform any assessments prior to the use of cDNA in library preparation?	N (Total 17)	Responses (%)
No quality checks are performed	8	47%
Yes, cDNA quantity is determined	3	18%
Yes, cDNA quantity is determined, and qPCR of a housekeeping gene is performed	5	29%
Other	1	6%
What controls are used in your NGS assay testing for gene fusions by RNA (cDNA) sequencing?	N (Total 17)	Responses (%)
Both and internal and external control	8	47%
An internal control only (e.g., housekeeping genes built into the assay)	4	24%
An external control only (e.g., a reference standard sample with known variants)	5	29%
What quality metrics are used for each sample/run to establish confidence that the result is valid (e.g., in a negative no-fusion sample)? (Choose all that apply)	N (Total 21)	Responses (%)
A minimum number of total reads after sequencing	14	67%
A minimum ct value from qPCR of a housekeeping gene	7	33%
When a fusion is detected on your NGS RNA assay, what quality metrics are used to determine that it is a true positive? (Choose all that apply)	N (Total 32)	Responses (%)
A minimum number of supporting reads spanning the fusion junction	13	41%
A minimum percentage of supporting reads spanning the fusion junction (e.g., fusion reads compared to total mapped reads)	7	22%
Expression imbalance assay, confirming ratio of 5′ and 3′ ends of the fusion (in amplicon methods)	5	16%
A minimum number of unique start sites (i.e., a subset of the unique reads that have unique fragment lengths)	4	13%
A minimum number of base pairs on either side of the breakpoint	2	6%
Other	1	3%
When a fusion is detected, what benchmarks are used to further inspect and confirm the fusion? (Choose all that apply)	N (Total 33)	Responses (%)
Fusions should be able to generate productive transcripts (in frame)	12	36%
Fusions are queried using databases	11	33%
Concordance of variants seen on DNA testing with RNA fusions in the same sample (e.g., for *MET* exon 14 skip variants)	7	21%
Novel fusions are confirmed using RNA with gene-specific primers flanking the fusion junction and confirmed by Sanger sequencing	1	3%
Other (report only validated fusions)	2	6%
Do you include an interpretive statement about fusions identified in your reports?	N (Total 17)	Responses (%)
Yes	17	100%
What information is used to produce an interpretive statement about the fusion to appear in reports?	N (Total 17)	Responses (%)
If the fusion is previously reported in databases or the literature or is novel	9	53%
The tumor histology, as reported in the accompanying pathology report	2	12%
Other (both databases/literature and tumor histology)	5	29%
Not applicable	1	6%
If a fusion corresponds to a variant seen on DNA testing on the same sample (e.g., *MET* exon 14) do you refer to both variants in your interpretive report?	N (Total 17)	Responses (%)
Yes	12	71%

No	5	29%
Do you include the following in your report?	N (Total 17)	Responses (%)
mRNA transcript references (e.g., NM numbers)	6	35%
Human reference genome (e.g., hg19)	4	24%
Other reference	1	6%
Not applicable	6	35%
What do you report regarding the number of reads that support the fusion?	N (Total 17)	Responses (%)
Do not report the number of reads seen with the fusion	6	35%
Report the number of reads containing the fusion	6	35%
Report the percentage of total reads containing the fusion	3	18%
Other (both total reads and percentage of reads, not yet determined)	2	12%
Where two fusions which are similar but differ by one exon [e.g., GeneX (exon 9)::GeneY (exon 14) and GeneX (exon 10)::GeneY (exon 14)] are observed, do you report both fusions or only the most abundant?	N (Total 17)	Responses (%)
Report only the most abundant fusion	10	59%
Report both fusions	5	29%
Other (have not yet encountered this scenario)	2	12%
Are the molecular lab reports consolidated into an overall pathology report?	N (Total 17)	Responses (%)
Yes, for all solid tumors	7	41%
Yes, for some solid tumors (i.e., for certain histologies)	4	24%
No, the molecular report is always released directly by the molecular lab	4	24%
Other	2	12%
Do you participate in any external quality assessment schemes for fusion detection by NGS?	N (Total 17)	Responses (%)
Yes, in a formal external quality assessment scheme	9	53%
Yes, in an informal quality assessment (e.g., interlaboratory comparison, intralaboratory comparison, blinded re-testing)	5	29%
No external quality assessment is performed	3	18%
